# Pre‐Torsion Tubular Metamaterials: Multi‐Effect Integration for Advanced Functional Applications

**DOI:** 10.1002/advs.202512564

**Published:** 2025-08-27

**Authors:** Xuegang Zhang, Jianfei Yin, Xin Ren, Jie Wu, Yang Wang, Xingchi Teng, Wei Jiang, Dong Han, Xihai Ni, Yi Zhang, Dianlong Yu, Jihong Wen

**Affiliations:** ^1^ College of Intelligence Science and Technology National University of Defense Technology Changsha 410073 China; ^2^ National Key Laboratory of Equipment State Sensing and Smart Support National University of Defense Technology Changsha 410073 China; ^3^ Center for Innovative Structures Nanjing Tech University Nanjing 211816 China

**Keywords:** auxetic tubular structure, compression‐torsion effect, multi‐effect, auxetic nail, pre‐torsion

## Abstract

Mechanical metamaterials continuously push the boundaries of mechanical properties far beyond conventional materials. However, a critical step toward the applications of metamaterials lies in combining multiple effects and functionalities into a single structure. Here, a pre‐torsion design paradigm is proposed to enable synergistic coupling of distinct mechanical properties. By incorporating such a paradigm into the auxetic tubular structures (ATSs), the robust and superior multi‐effect integration (MEI) performance consisting of compression‐torsion and auxetic effect under both axial compression and tension is achieved, and the underlying mechanisms are investigated numerically and experimentally. To further demonstrate their benefits in practical engineering, pre‐torsion auxetic nails are fabricated based on this design philosophy. Driving‐in tests have confirmed their MEI property and manifested extraordinary easy‐insertion and damage‐free performance. Compared to conventional nails, the pre‐torsion nail demonstrates a 52% reduction in energy consumption during wood penetration, along with a 27.1% decrease in initial peak force. This work bridges a crucial gap toward the applications of mechanical metamaterials and can be extended to various engineering scenarios for the design of functional structures and devices.

## Graphical abstract

Through a pre‐torsion design paradigm, the authors design and fabricate a class of multi‐effect mechanical metamaterial that synergistically integrates compression‐torsion and auxetic behaviors over an extensive strain range, and their formation mechanisms are quantitatively analyzed and elucidated via experiments and finite element methods. The superior performance of such metamaterials in practical applications is demonstrated by integrating them into the fastener design, highlighting their enormous potential in engineering.

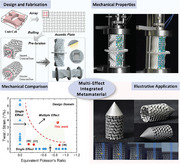

## 1 Introduction

Mechanical metamaterials have revolutionized conventional notions of engineering materials and structures by enabling a range of exotic mechanical properties.^[^ ^1^, ^2^, ^3^, ^4^, ^5^ ^]^ These unique characteristics stem from the carefully orchestrated mesoscopic structures rather than the constituent materials. The boundless design flexibility allows unprecedented functionalities such as counter‐intuitive deformation,^[^ ^6^, ^7^, ^8^ ^]^ elastic wave modulation,^[^ ^9^, ^10^, ^11^ ^]^ and a combination of lightweight and high‐strength characteristics.^[^ ^12^, ^13^, ^14^, ^15^, ^16^, ^17^ ^]^

Compression‐torsion metamaterials (CTMs), a recently developed subset of mechanical metamaterials, have garnered significant research interest due to their ability to transcend the limitations of Cauchy's relations in continuum mechanics by exhibiting the coupling of compressive and torsional deformations.^[^ ^18^ ^]^ Frenzel et al. initially designed a class of 3D metamaterials using tetrachiral lattices to demonstrate the twisting motion under axial compression.^[^ ^19^ ^]^ Notably, this compression‐torsion coupling exhibits similarities to the well‐known Poynting effect observed in mechanical systems.^[^ ^20^ ^]^ The Poynting effect refers to the shear‐induced or torsion‐induced normal stress in the direction perpendicular to the shear (torsion) plane.^[^ ^21^ ^]^ Although both effects demonstrate coupling between torsion/shear and compression, they are fundamentally distinct phenomena with different driving mechanisms.^[^ ^22^ ^]^ Recent investigations into the Poynting effect have primarily centered on characterizing the nonlinear Poynting modulus, mitigating torsional buckling instabilities, and the applications in soft matter.^[^ ^21^, ^22^, ^23^ ^]^

**Figure 1:**
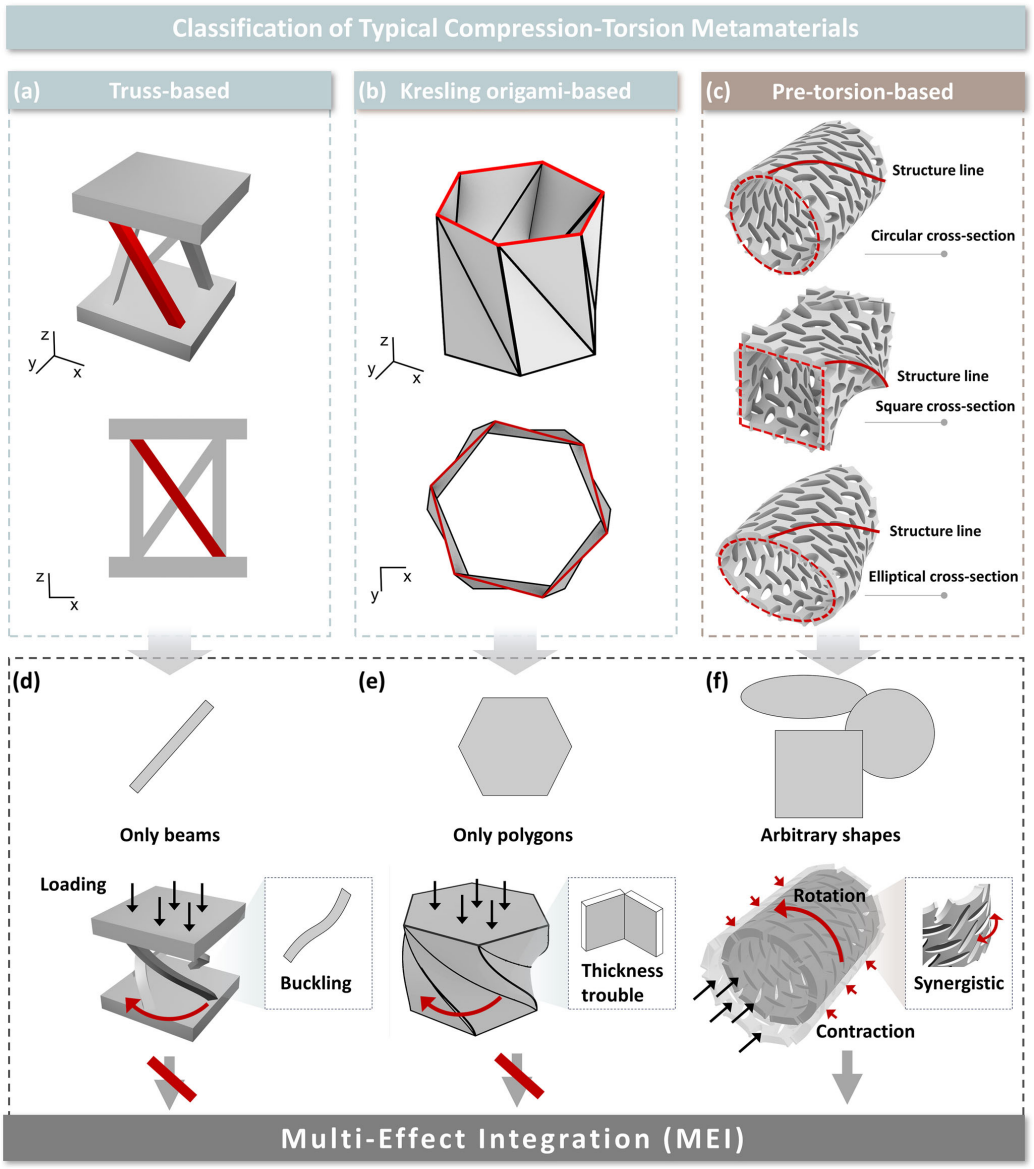
Classification of typical CTMs. a) Truss‐based, b) Kresling origami‐based, and c) pre‐torsion‐based CTMs proposed in this work. d) Truss‐based CTMs consist of diagonally oriented beams that form the body of the structure; in this case, the bending deformation of the beams induces the structure to rotate. Buckling occurs when the critical load is reached. Moreover, truss‐based CTMs struggle to achieve MEI, as their configuration is inherently designed for compression‐torsion effects. e) Kresling origami‐based CTMs arrange diagonally aligned mountain and valley crease lines on a plate, which undergo rotational deformation along the creases when compressed. But one may encounter difficulties in folding thick plates and integrating auxetic effects effectively. f) In this work, we present a new design paradigm of CTMs for tubular/columnar structures with arbitrary cross‐sections. Torsion is generated along the structural line during compression or tension. Such a design overcomes the problems mentioned above and is promising to integrate multiple effects.

Recent developments in CTMs have produced diverse architectures including truss‐based,^[^ ^24^, ^25^, ^26^ ^]^ shell‐based,^[^ ^27^ ^]^ origami‐based,^[^ ^28^, ^29^ ^]^ kirigami‐based,^[^ ^30^ ^]^ and topology‐optimized structures,^[^ ^31^, ^32^ ^]^ etc. Among these, truss‐based and origami‐based configurations, as illustrated in **Figure** **1**a,b, have emerged as the most prevalent due to their design versatility and well‐characterized mechanical properties. Specifically, truss‐based CTMs typically consist of eccentric rods arranged to form a torsional system. When subjected to load, these diagonal rods undergo buckling, thereby inducing torsional deformation. Origami‐based CTMs, on the other hand, can be designed using different folding patterns, such as the Kresling pattern, where the diagonal folds alter force transmission paths to generate torsion. Unlike conventional rule‐based design approaches, recent topology optimization methods for CTMs enable the generation of complex, organic geometries that maximize compression‐torsion coupling within given constraints, potentially achieving superior mechanical performance.^[^ ^31^, ^32^ ^]^ While significant progress has been made in realizing compression‐torsion coupling, most prior research has focused on enhancing the effect, typically quantified by the twisting angle per unit strain.

In CTMs, the compression‐torsion effect originates from chiral asymmetric deformation patterns.^[^ ^33^ ^]^ The inclusion of chirality in metamaterials can further impart enhanced mechanical properties, such as a negative Poisson's ratio, which can benefit MEI design. However, simultaneously realizing and tuning both compression‐torsion coupling and auxetic behavior, termed the “deep integration of advanced functionalities”, remains a challenging endeavor.^[^ ^34^ ^]^ This integration is crucial for advancing the practical applications of metamaterials.^[^ ^35^ ^]^ To date, only limited studies have explored the coupling of multiple effects in metamaterials.^[^ ^36^, ^37^ ^]^ Recent work on porous structures has identified the presence of multiple mechanical behaviors,^[^ ^38^ ^]^ yet the engineering applications of these coupled effects in advanced structures and materials need further exploration.

In this paper, we propose a pre‐torsion design paradigm that allows for the seamless integration of multiple mechanical effects based on the auxetic tubular structure (ATS) (Figure 1c). Experimental and numerical analyses have shown that the pre‐torsion ATSs not only sustain the auxetic effect but also exhibit robust compression/tension‐torsion effect even under large deformation. Our proposed pre‐torsion design paradigm effectively addresses the fundamental limitations of conventional CTM approaches. While truss‐based CTMs exhibit constrained design flexibility, typically limited to a single compression‐torsion mode while suffering from unpredictable buckling instabilities in their diagonal structural elements (Figure 1d);^[^ ^39^, ^40^, ^41^, ^42^ ^]^ origami‐based configurations face inherent stability challenges when implemented with thick plates due to physical interference during folding, rendering them unsuitable for MEI designs (Figure 1e).^[^ ^29^, ^43^ ^]^ The pre‐torsion paradigm increases the flexibility of the design while guaranteeing structural stability in the circumferential direction (Figure 1f). Numerical simulations further demonstrate that stable MEI can be maintained with increased wall thickness. Moreover, as a practical demonstration of the advantages of MEI in engineering, we design and fabricate pre‐torsion auxetic nails. Experimental results show that these pre‐torsion auxetic nails exhibit both the compression‐torsion effect and the auxetic effect. The driving‐in of such nails requires much less thrust and poses no damage to the object compared with normal nails and auxetic nails. The pre‐torsion auxetic nail outperforms conventional nails by reducing energy consumption during wood penetration by 52%, while simultaneously lowering the initial peak force by 27.1%. The pre‐torsion design paradigm potentially paves the way for new avenues in both applied and fundamental research on CTMs.

## 2 Results and Discussion

### 2.1 Pre‐Torsion Design Paradigm

**Figure 2:**
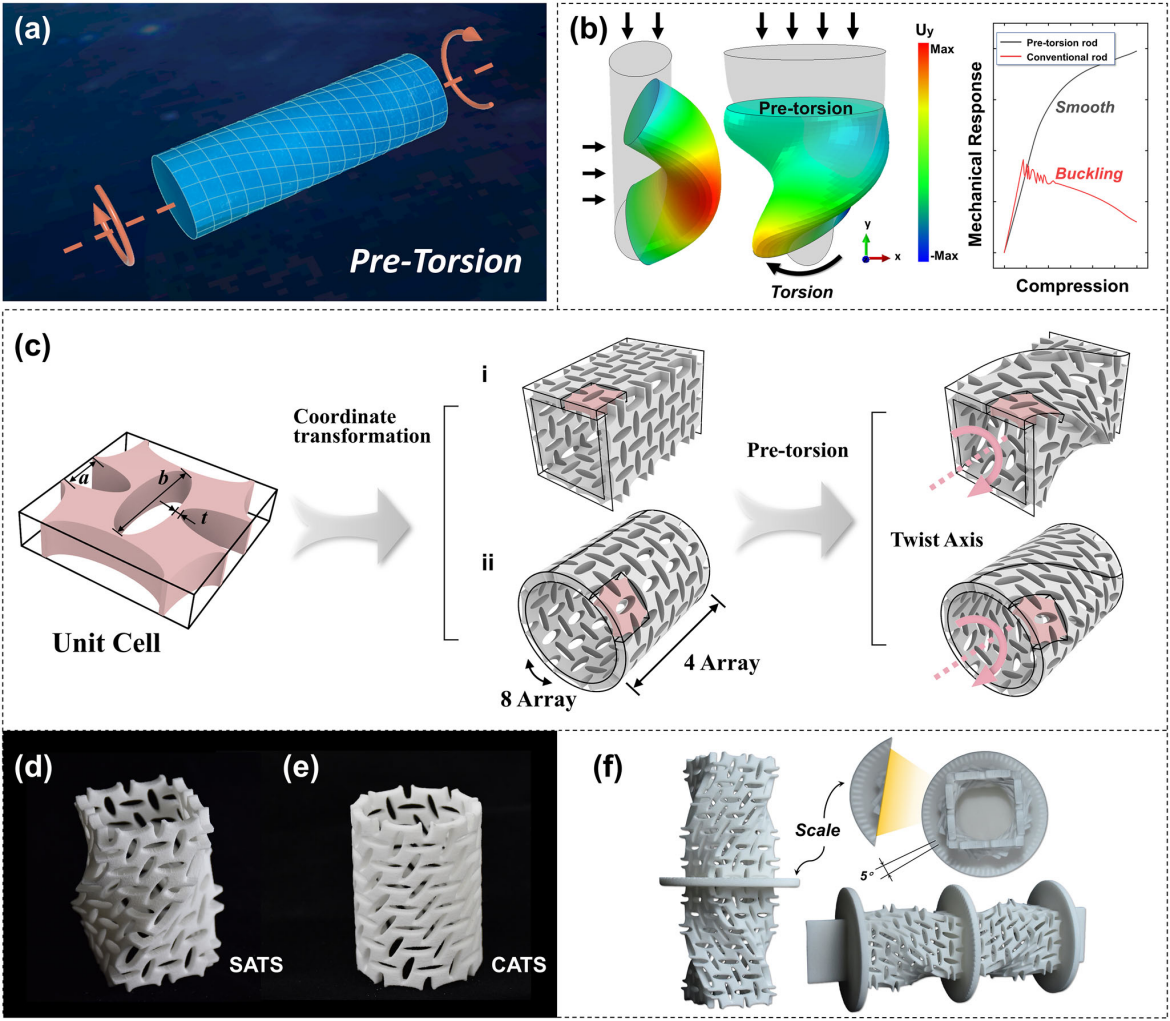
Pre‐torsion design paradigm and preparation of test samples. a) The design concept of pre‐torsion, where torsion is applied at the end of the structure to create torsional deformation. b) Mechanical response under axial compression of a rod with elliptical cross‐section and the same rod with pre‐torsion deformation. During compression, buckling occurs for the normal rod, while the pre‐torsion rod exhibits a smooth deformation and is endowed with additional torsional freedom. c) Pre‐torsion design procedure for ATSs. Step I: a 2D auxetic perforated plate is designed. Step II: the 3D tubular structures with circular and square cross‐sections are generated. Step III: Pre‐torsion angular deformation of 90° is applied to the tubular structures. d,e) Pre‐torsion ATSs are manufactured using 3D printing with thermoplastic polyurethane (TPU) materials. f) Compression and tension samples are designed with double‐layer mirror symmetry configuration allowing direct measurement of the torsional angles via the scale lines carved on the middle disc with each increment representing 5°.

**Figure** **2**a illustrates the design principle of pre‐torsion, wherein a torsional deformation is pre‐applied at both ends of the structure. Notably, in our design, no residual stress distribution remains within the structure following the pre‐deformation process.^[^ ^44^, ^45^ ^]^ A rod with pre‐torsion deformation under compression has revealed that not only is the compressive instability effectively mitigated without adding mass to the structure, but also a compression‐torsion characteristic in mechanical response is observed (Figure 2b).

In this work, we incorporate such a paradigm into ATSs to develop the MEI design. ATSs, as a particular branch of auxetic structures, have garnered significant research interest recently aimed at utilizing the desirable auxetic behavior for functional applications.^[^ ^46^ ^]^ The selection of ATS as the candidate for our blueprint is motivated by three key considerations: i) The ATS exhibits lightweight characteristics while demonstrating exceptional performance in load‐bearing and energy absorption. ii) Under axial compression, ATS demonstrates radial auxetic behavior that shares compatible loading conditions with the compression‐torsion coupling effect, making it promising for achieving our envisioned multi‐effect integration. iii) Compared to alternative structural configurations, the tubular geometry proves more suitable for implementing the pre‐torsion design paradigm.

The procedure for generating pre‐torsion auxetic tubular structures is illustrated in Figure 2c. A 2D auxetic perforated plate is designed and then arrayed and rolled to form the 3D ATS using the coordinate transformation method.^[^ ^47^ ^]^ The cross‐section shape of the ATS can be practically arbitrary, and in this work, we use two typical configurations for analysis: a square cross‐section auxetic tubular structure (SATS) and a circular cross‐section auxetic tubular structure (CATS).^[^ ^48^ ^]^ Detailed design parameters for these structures are described in Figures S1 and S2 (Supporting Information). A pre‐torsion angular deformation of 90° is applied to both SATS and CATS. Note that the cross‐section of the SATS undergoes continuous rotation along the longitudinal axis of the tube, while for CATS, only the pores on the tube wall change due to its consistent curvature. As a result, SATS experiences cross‐sectional deformation, while CATS preserves its original morphological profile.

The pre‐torsion ATSs (Figure 2d,e) are fabricated using 3D printing (see Methods). The test samples are prepared following a similar configuration to that used by Frenzel et al.,^[^ ^19^ ^]^ in which two layers of ATSs with mirror symmetry are connected via a middle disc. This setup ensures that the rotational freedom of the disc is fully released, and the overall torque of the sample is zero. To measure the twist angle *θ*, scale lines are incorporated on the middle disc, with each increment representing 5 degrees (Figure 2f).

### 2.2 Multi‐Effect Mechanical Properties

**Figure 3:**
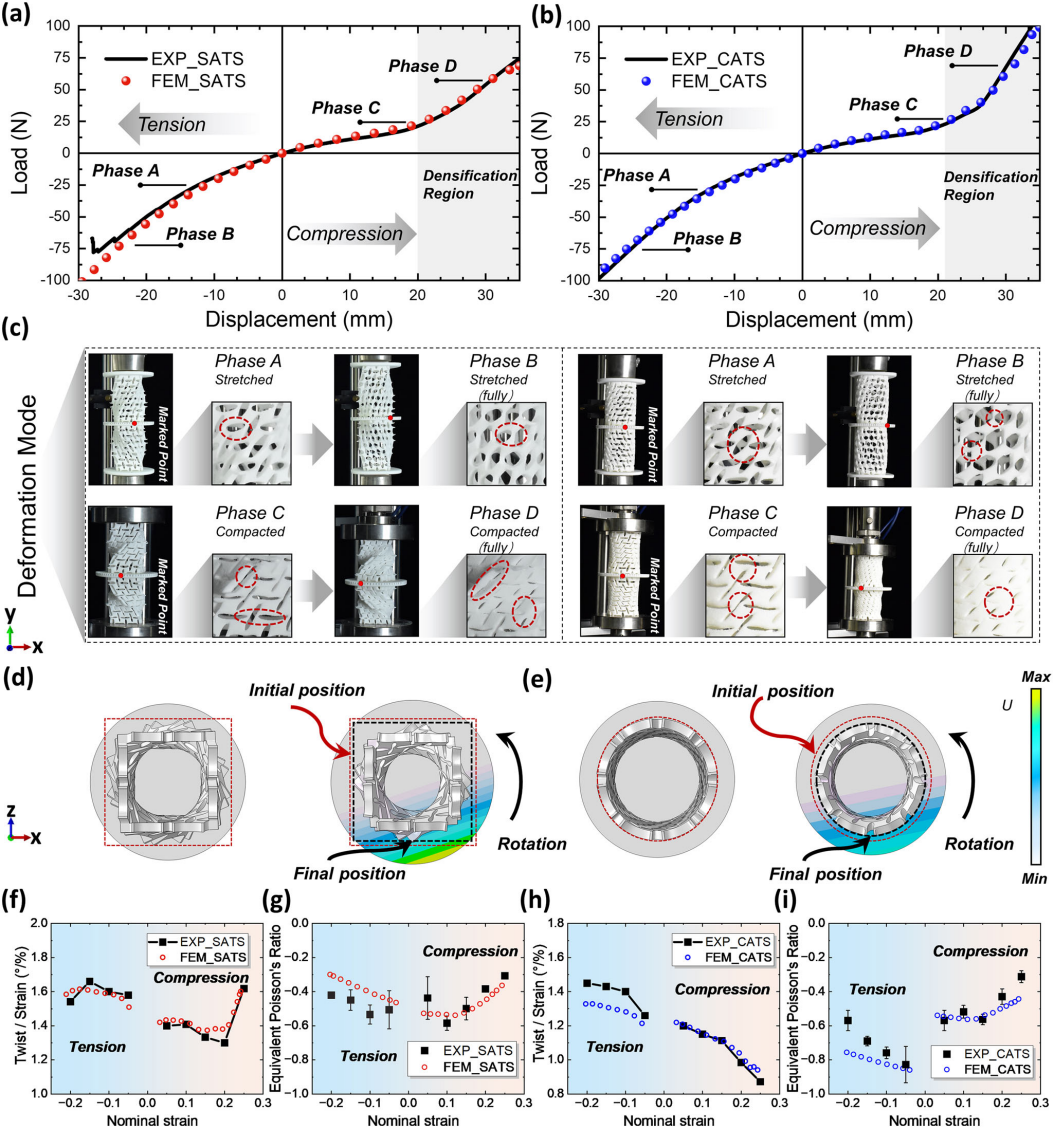
Mechanical behavior of pre‐torsion ATSs under compressive and tensile loading. a,b) Load‐displacement curves for compression and tension tests obtained from experiments and FEM. The grey box represents the densification region where the stiffness of the structure increases steeply. c) The deformation modes of the two pre‐torsion tubular structures in tension and compression. Phase A to phase B represents the tensile process, while phase C to phase D represents the compressive process. Stretched and compacted states of the holes in the tube wall are shown in the enlarged view. d,e) The top view during compression tests illustrates the auxetic effect by comparing the boundary before (red dashed line) and after compression (black dashed line). f,g) Quantified torsion angle and equivalent Poisson's ratio for pre‐torsion SATS. h,i) Quantified torsion angle and equivalent Poisson's ratio for the pre‐torsion CATS.

The mechanical properties of the pre‐torsion ATSs are evaluated using experiments and the finite element method (FEM), and the results obtained from both methods are in good agreement (see Methods, and the boundary conditions in FEM are given in Figure S4, Supporting Information). Both experimental and FEM results reveal the disparity in mechanical behavior for tension and compression, which has often been overlooked in previous studies.^[^ ^49^, ^50^ ^]^ The load‐displacement curves in **Figure** **3**a,b show that the mechanical responses of compression and tension loading are similar for both types of pre‐torsion ATSs, exhibiting smooth responses without obvious yield, which is associated with the hyperelastic constitutive model. Under compression, the samples reach densification at ≈0.25 nominal strain, at which point the pores of ATSs are fully compacted (Figure S5a,c, Supporting Information), while under tension, they are prone to fracture under larger deformation. Therefore, the mechanical behaviors under tension are presented only up to 0.2 strain (Figure S5b,d, Supporting Information). In the densification region, a notable difference in mechanical responses is observed between SATS and CATS samples. The SATS sample continues to rotate along the convex structure line (mentioned in Figure 1) after densification, while the CATS sample maintains a uniform alignment with the structure line, resulting in a reduced compression‐torsion effect and a substantial enhancement in its load‐bearing capability.

Figure 3c displays the deformation modes corresponding to the phases A‐D described in Figure 3a,b under tension and compression. Both STAS and CATS exhibit circumferential expansion and counterclockwise rotation around the y‐axis in tension and circumferential contraction and clockwise rotation in compression. Phase A is in the initial tensile state, and the tubular structure continues to rotate and expand. Then, the ligament is fully stretched and reaches the ultimate strength of the base material (phase B). At this point, the holes in the wall of the tube are fully expanded, achieving the maximum negative Poisson's ratio and torsional angle. If loading continues, a fracture occurs. At phase C under compression, several holes begin to compact, causing the stiffness to increase, and the load‐displacement curve transitions into the densification region. The auxetic effect begins to weaken, which is manifested as a decrease in the negative Poisson's ratio value. However, the compression‐torsion behavior persists, with the tubular structure continuing to rotate along the structure line. In phase D, the holes become fully compacted. The structure morphs into a solid tube, directly transferring the load, which leads to the disappearance of the MEI. The middle discs exhibit a significant twist during tension and compression, as indicated by the movement of the red marks, which confirms the compression/tension‐torsion coupling effect. Furthermore, the auxetic effect has been identified through the observation of distinct lateral expansion under tension and corresponding contraction during compression.

From the top view (*x*‐*z* plane) in compression tests, a shrinkage of the external boundary is found for both CATS and SATS samples as a validation of the auxetic effect (Figure 3d,e). In addition, the displacement in the *x*‐direction of the central disc of the two pre‐torsion ATSs is monitored (cloud map in the top view), demonstrating a close association with the torsional angle, as described below.

Both types of pre‐torsion ATSs exhibit larger torsional angles under tensile loading than compressive loading, with a maximum twisting angle of ≈1.66° per strain observed for the SATS sample under tension (Figure 3f). Regarding the auxetic behavior, the equivalent Poisson's ratios of the samples are calculated using the method illustrated in Figure S6 (Supporting Information), with the results shown in Figure 3g,i. Here, the equivalent Poisson's ratio is defined as the average degree of contraction or expansion of the tube diameter of the pre‐torsion ATS in axial tension or compression. Due to symmetry, only half of the structure is taken for calculations. Notably, the CATS demonstrates a maximum negative Poisson's ratio under tension of ≈−0.82 (Figure 3i). The mechanical tests have validated that the pre‐torsion design of ATSs not only preserves the auxetic effect but also introduces tension/compression‐torsion behavior, thereby fulfilling the intended MEI objective.

**Figure 4:**
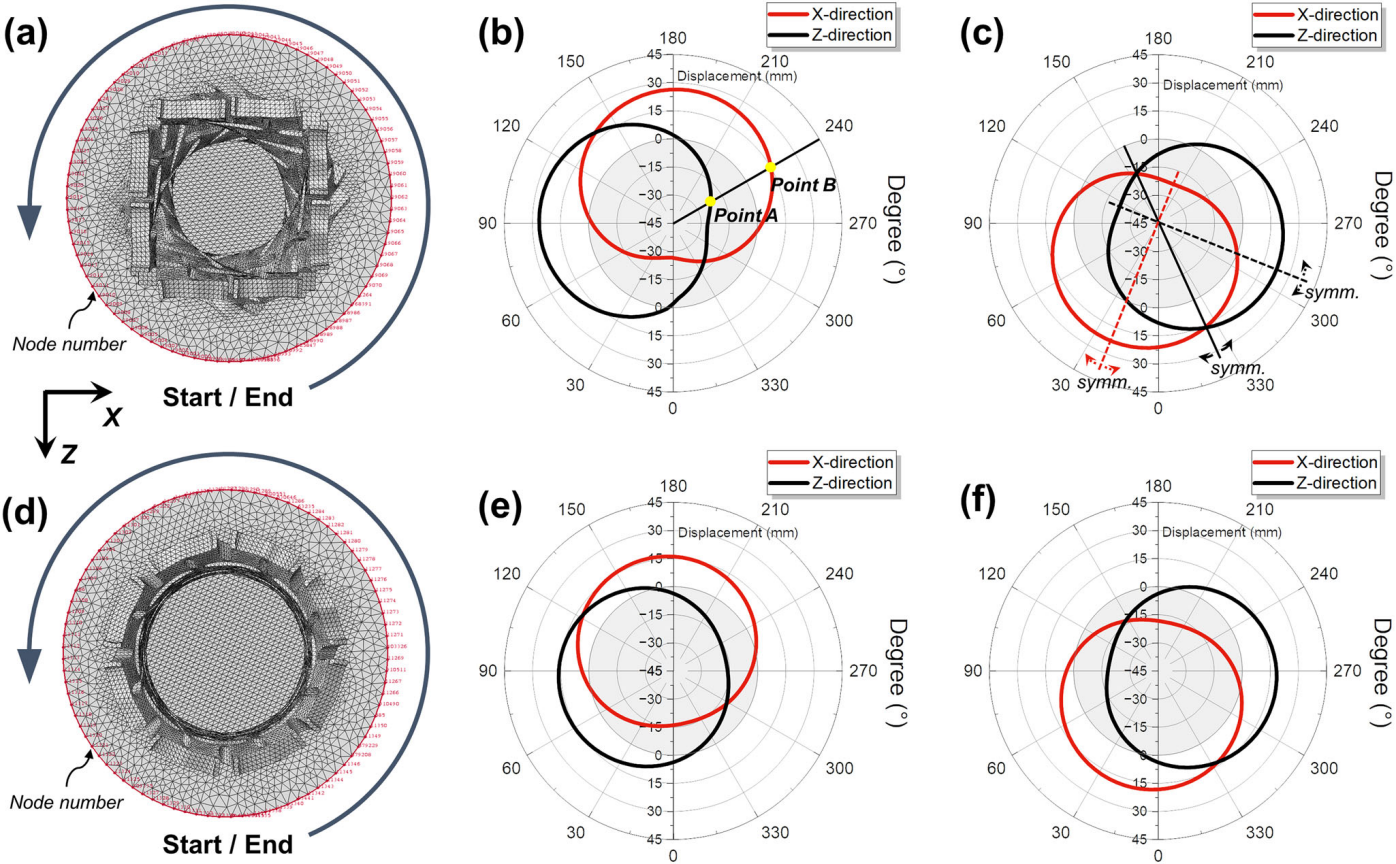
Extraction of the nodal displacements in FEM. a) Nodes along the edge of the middle disc for SATS are marked by node numbers. b,c) The corresponding nodal displacement radar plots in *x*‐ and *z*‐direction under compression and tension. The 0°/360°position is labeled as ‘Start/End’ in Figure 4a. d‐f) Nodes for CATS and its corresponding nodal displacement plots. The torsional angle of the middle disc can be calculated from the translational displacements.

We examine the torsional characteristics of the pre‐torsion ATSs under compression and tension loading (**Figure** **4**). The translational displacements in the *x*‐*z* plane of the circumferential nodes at the edge of the middle disc are recorded in FEM, from which the torsional angle at any point of the disc can be calculated by

(1) $$\theta =2/\pi \times 180{}^{\circ}\times \;\arcsin\left(\sqrt{\left(A^{2}+B^{2}\right)/84}\right)$$

where A and B are displacements in *x*‐ and *z*‐direction, as illustrated in Figure 4b. As a note, displacement in the *x*‐direction and *z*‐direction is symmetrical on the disc (Figure 4c). The trajectories of the circumferential nodes in the *x* and *y* directions are also symmetric along the intersection of the curves, and the twist angle *θ* is consistent at any angle within the circumference. It is illustrated that the disk does not affect the torsional effect of the whole system.

### 2.3 Mechanism of MEI and Parameter Effects

**Figure 5:**
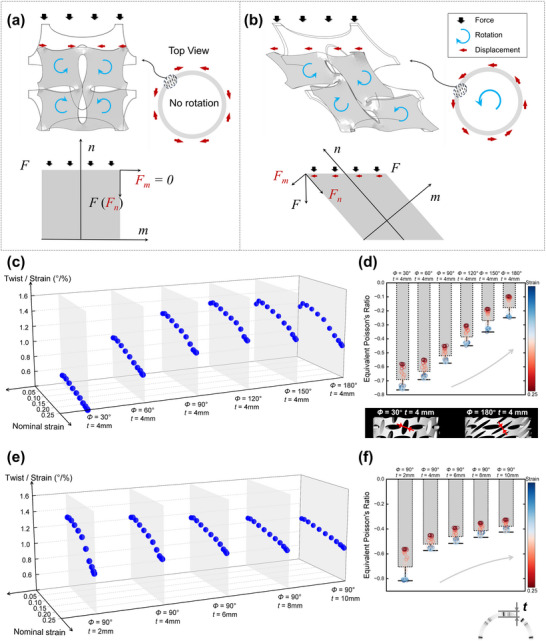
Force mechanism analysis and parametric effects of MEI. a) Deformation pattern under compressive loading for the APP, where a clear auxetic effect can be observed. No rotation is generated in the circumferential direction of the tubular structure. b) Deformation of the pre‐torsion APP allows the emergence of lateral displacement while maintaining the auxetic effect under compression. Rotation is generated in the circumferential direction of the tubular structure. In the force diagram for APP and pre‐torsion APP, the latter produces a diagonal component force *F_m_* under the axial force *F*, which is responsible for the macroscopic compression‐torsion effect. c,d) Quantified torsion angle of the pre‐torsion SATS with different pre‐torsion angle *Φ* (from 30° to 180° at 30° intervals). e,f) Quantified torsion angle and equivalent Poisson's ratio for pre‐torsion SATS with different wall thicknesses t (from 2 mm to 10 mm at 2 mm intervals).

To elucidate the mechanism of MEI in the pre‐torsion ATSs, we perform force analysis on the auxetic perforated plate (APP) that constitutes the ATSs. Generally, a tubular structure can be simplified as the unfolded planar structure under compression or simple shear with deformation compatibility requirements.^[^ ^22^ ^]^ **Figure** **5**a,b illustrates the deformation characteristics for the normal APP in comparison with the pre‐torsion APP under compressive loading. A clear auxetic effect is observed in both cases, primarily due to the buckling‐induced rotation of the unit cells.^[^ ^51^, ^52^, ^53^ ^]^ For the normal APP, the symmetrical deformation cancels out the rotational displacement, resulting in negligible macroscopic torsion (Figure 5a). By contrast, the pre‐torsion treatment disrupts this symmetrical deformation under compression. As a result, the axial force generates a lateral shear component, leading to a macroscopic rotational displacement along the tube axis. From Figure 5b, the pre‐torsion APP generates a non‐axial diagonal component of the force under axial compressive loading relative to the normal APP, which gives it an additional lateral translational degree of freedom (torsion in the tubular structure). Therefore, it can be demonstrated that the structures designed with pre‐torsion can realize compression‐torsion effects while maintaining the negative Poisson's ratio effect.

For the pre‐torsion tubular structure with a circular cross‐section, we analyze the effect of the pre‐torsion angle *Φ* and the wall thickness *t* on the torsion angle and the equivalent Poisson's ratio under compressive loading. An increase in the pre‐torsion angle enhances the compression‐torsion effect of the tubular structure, reaching a maximum when *Φ* equals 120° (at 0.031 strain, with a torsion angle of 1.34°/%). Beyond this point, the compression‐torsion effect gradually weakens, as illustrated in Figure 5c. Note that we demonstrate that increasing the height of the pre‐torsion tubular structure amplifies the compression‐torsion effect (refer to Figure S7, Supporting Information).

The auxetic effect weakens as the pre‐torsion angle *Φ* increases, with the negative Poisson's ratio reaching −0.77 at a pre‐torsion angle of 30° (Figure 5d). At 180°, the maximum equivalent negative Poisson's ratio decreases to −0.25. This occurs because the elliptical holes in the tube wall become more inclined and elongated as the pre‐torsion angle increases, which enhances the compression‐torsion effect but reduces the auxetic effect.

Figure 5e depicts that increasing the wall thickness of the pre‐torsion tubular structure reduces both the compression‐torsion and auxetic effects. The most significant values of torsion angle and negative Poisson's ratio are observed with a wall thickness of 2 mm, which can be explained as the thin‐walled tubes are more easily deformed, while the increase in wall thickness restricts the overall deformation of the tubular structure (Figure 5f). In addition, we investigate the effect of the axial ratio of the elliptical perforations on the mechanical properties of the pre‐torsion ATSs (Figure S8, Supporting Information). As the perforation approaches a circular shape, the pre‐torsion ATS begins to buckle at the wall, and the auxetic effect vanishes. However, the compression‐torsion effect persists regardless of the axial ratio, demonstrating the robustness of our design paradigm.

### 2.4 Mechanical Comparison

**Figure 6:**
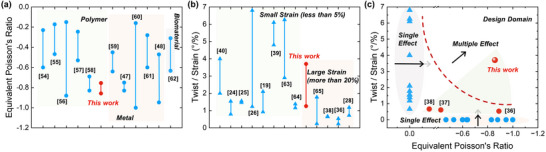
Comparison with the mechanical performance between the proposed pre‐torsion ATSs and metamaterials reported in previous literature. a) Equivalent Poisson's ratio. The vertical lines denote the range of values observed, and color blocks represent different constituent materials of the ATSs: light green for polymer material, light orange for metal‐based structures, and gray for biological material. b) Torsion angles of compression‐torsion effect. The color blocks indicate the strain range; light green represents less than 5%, and light orange indicates greater than 20%. c) A comprehensive comparison. Light green areas represent structures with multiple effects, while gray and light orange areas indicate structures with a single effect.

We now compare the equivalent Poisson's ratio and compression‐torsion effect of the pre‐torsion ATSs with the structures reported in previous studies, including various ATSs and compression‐torsion structures (**Figure** **6**).

In terms of the auxetic effect, research on ATSs can be mainly categorized as polymers^[^ ^54^, ^55^, ^56^, ^57^, ^58^ ^]^ metals,^[^ ^47^, ^48^, ^59^, ^60^, ^61^ ^]^ and biomaterials^[^ ^62^ ^]^ based on their constituent material. Note that we set two conditions in filtering previous works to ensure a fair comparison: i) nominal strain greater than 10% and ii) exclusion of truss‐based auxetic tubular structures whose relative density is very low. As depicted in Figure 6a, metal‐based ATSs generally exhibit a greater auxetic effect than polymer‐based and biomaterial‐based ATSs, owing to their high ductility. Notably, the negative Poisson's ratio of our proposed pre‐torsion ATS reaches −0.86, which excels among polymer‐based ATSs. This demonstrates that the pre‐torsion design does not compromise the auxetic behavior of the original structure.

As for the compression‐torsion effect, previous research typically focuses on small deformations (i.e., less than 5% strain).^[^ ^19^, ^24^, ^25^, ^26^, ^39^, ^40^, ^63^, ^64^ ^]^ In these cases, truss‐based CTMs can achieve a large compression‐torsion effect (6.8°/% or more) due to the low bending stiffness of slender beams, which are prone to deformation in the elastic range.^[^ ^26^ ^]^ However, such designs are susceptible to instability or fracture when the deformation is over the elastic range (over than 20% strain), hence most studies have limited to the small deformation range (Figure 6b). Conversely, in the case of large deformation,^[^ ^28^, ^36^, ^38^, ^65^ ^]^ the proposed pre‐torsion ATSs have demonstrated the most stable and highest compression‐torsion effect, reaching 3.7°/%. Furthermore, the performance can be enhanced by increasing the number of layers of the ATS (Figure S7, Supporting Information).

As shown in Figure 6c, a comprehensive comparison of the mechanical performance among the structures mentioned above has demonstrated the advantages of the pre‐torsion ATSs. Though previous works may excel in one specific aspect, the MEI performance cannot be observed due to the contradictions in design principles.^[^ ^66^ ^]^ In contrast, the proposed structures provide superior overall performance and a broader performance range. Among a few works that considered MEI, this work also significantly enhances the compression‐torsion effect and auxetic effect.

### 2.5 Demonstrative Application of the Pre‐Torsion ATS

**Figure 7:**
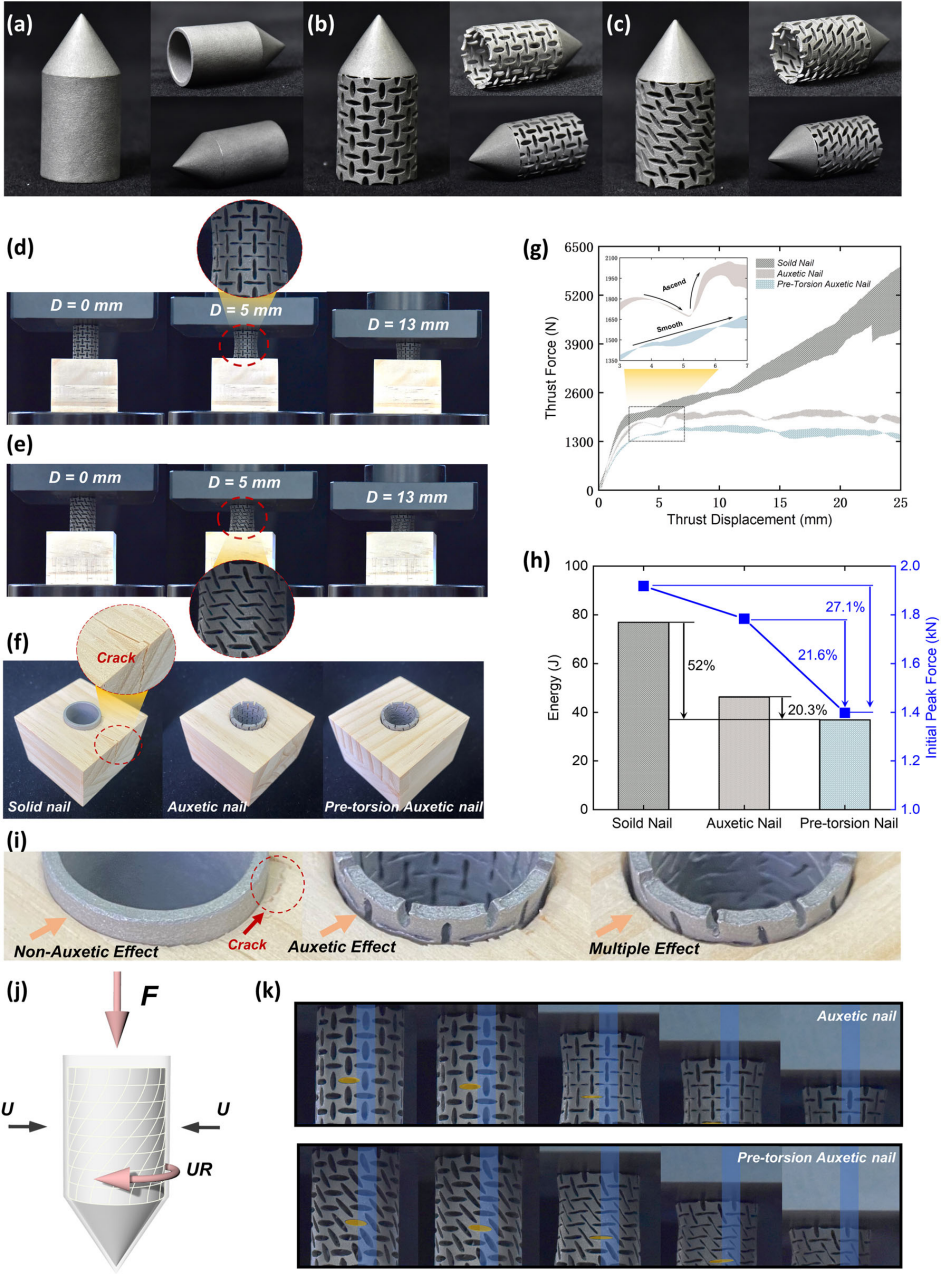
Tests of nails being driven into wooden blocks. a–c) Photos of the solid nails, auxetic nails, and pre‐torsion auxetic nails. d,e) Driving‐in test of the auxetic nail and pre‐torsion auxetic nail. In both cases, a clear auxetic effect can be observed as the shanks of nails experience lateral contraction during compression. f) Nailed wooden blocks after the test. Cracks appear in the solid nail during the test, while the other two remain intact. g) Thrust force‐displacement curves for three types of nails. h) Energy and initial peak force for nails thrusting into wooden blocks. i) Enlarged figures of the nailed blocks after the test. j) Push‐in schematic diagram of the pre‐torsion auxetic nail. k) Snapshots during compression where the compression‐torsion effect is monitored by marking a specific hole on the shank of the nail (yellow block). The blue block serves as a stationary reference.

As an illustrative application, we implement the pre‐torsion design in nails.^[^ ^67^ ^]^ Three types of tubular hollow nails are fabricated using 3D printing: solid, auxetic, and pre‐torsion auxetic nails (**Figure** **7**a–c). The detailed design and experimental methods are described in Figure S9 (Supporting Information). Note that we have ensured that the dimensions of the three nails are identical; the difference is in the construction of the shanks of the nails. For the auxetic and pre‐torsion versions of the nails, their mass is exactly the same, as the pre‐torsion design does not change the mass. When the auxetic and pre‐torsion auxetic nails are driven into the wooden blocks, both nails demonstrate evident negative Poisson's ratio effect (Figure 7d,e). For the solid nail driving‐in test, a crack occurs at the edge of the wood block due to the violent squeezing force, while the wooden blocks for auxetic and pre‐torsion auxetic nails remain intact (Figure 7f–i). This is because the axial force induces the auxetic effect within the shank of the nail, which diminishes the contact area between the nail and the wooden block. As a result, the squeeze force and friction can be notably mitigated.

In the force‐displacement curve (Figure 7g), the frictional force of solid nails intensifies progressively as the contact area expands during the process of insertion, thereby causing the load to rise rapidly. In addition, a steeper drop of the force curve results from the damage to the wooden block. On the contrary, both auxetic nails experience lower loads and are more easily driven into the wooden block. It is also observed that the load of the auxetic nail exhibits a snap‐through (Figure 7g, the partially enlarged figure) during insertion. This is due to the rise of load after the densification of the shank of the nail. In contrast, the pre‐torsion auxetic nails did not experience such load fluctuations during insertion due to the compression‐torsion effect (Figure 7j) so the driven‐in process is more stable and smoother than the normal auxetic nail.

Quantitative comparisons show that the total energy consumption of the pre‐torsion auxetic nails for insertion into the wood block is reduced by 52.0% compared to the solid nails and 20.3% compared to the auxetic nails. In addition, the initial peak force is reduced by 27.1% and 21.6% compared to solid nails and auxetic nails, respectively (Figure 7h). This enhancement originated from the coupling of multiple effects (Figure 7j).

Figure 7k visualizes the process of driving the nail into the block and traces the movement of a specific hole on the shank of the nail marked by a yellow block. For the auxetic nail, the marked hole moves straight down along the axis of the nail, while for the pre‐torsion auxetic nail, the marked hole gradually rotates and moves horizontally into the blue region. Note that the blue area marked in Figure 7k is fixed as a visual reference. Due to the significant boundary effect, the twisting angle is difficult to reach as high as the results in Section 2.2. The results of the tests have demonstrated that the incorporation of the compression‐torsion effect in the design of nails can contribute to overcoming the resistance of objects to the nail and thus facilitate a smoother and easier driving‐in process.

In this section, we have successfully validated the rationality of MEI through nail driving‐in tests, which are inaccessible by conventional designs. In fact, the pre‐torsion design paradigm remains open in the design of various functional parts such as fasteners.

## 3 Conclusion

In summary, we propose a pre‐torsion design paradigm that prevents buckling while enabling synergistic integration of multiple mechanical effects. The pre‐torsion design is then integrated into ATSs, and a combination of compression/tension‐torsion effect and auxetic effect is demonstrated harmoniously, up to 3.7°/% and ‐0.86, respectively. Mechanisms attributed to torsion of ATSs from the diagonal force component under compressive loading, which is caused by the pre‐torsion design. Then, we find that the proposed structure has robust and superior multiple effects compared to previous works that may excel in one specific aspect through a comprehensive comparison. To demonstrate the advantages of MEI in engineering applications, we design and fabricate a novel pre‐torsion auxetic nail. Compared to conventional nails, the pre‐torsion nail demonstrates a 52% reduction in energy consumption during wood penetration, along with a 27.1% decrease in initial peak force.

Moreover, we envision that the design method in this study will bring new opportunities in the application of elastic wave manipulation.^[^ ^68^, ^69^ ^]^ We investigate the band structure of the pre‐torsion ATSs, illustrated in Figure S10, Supporting Information. Remarkably, the pre‐torsion ATS can generate low frequency bandgaps at 57–75 and 148–160 Hz, suggesting their potential in elastic wave control, which gives a bonus point to the multi‐effect integration design.

Overall, this research not only underscores the superiority of multiple effects integration but also offers new insights into the construction of advanced structures/materials.

## 4 Experimental Section

### Fabrication of Specimens

To produce the pre‐torsion auxetic tubular structures, the 3D printer Farsoon‐104A was utilized, employing selective laser sintering (SLS) technology. The base material is the 95A Thermoplastic Polyurethane Elastomer (TPU) with the density of 1100 kg m^−3^. During the manufacturing process, a layer thickness of 100 µm is set, and the melting point of the 95A TPU powder is 122 °C. Five dumbbell‐shaped specimens are manufactured according to standard ISO‐37: 2017 to test the mechanical properties of the materials (see Figure S3, Supporting Information).^[^ ^70^ ^]^ As a note, there are some differences between the tensile and compressive samples. To meet the requirements of tensile tests, we set up plates for clamping at the ends of the tensile specimens, which affect the deformation of the boundaries, but have little effect on the mechanical behavior.

### Compression/Tension Test

To evaluate the mechanical behavior of the pre‐torsion auxetic tubular structures, all samples are tested under uniaxial tension and uniaxial compression with a speed of 2 mm min^−1^, which corresponds to a quasi–static loading process. The disc is marked for easy recording. The loading process is recorded by a camera (Canon EOS 850D), and the compression/tension torsion effects and negative Poisson's ratio effects are calculated from the movement of the marked points (see Figure S6, Supporting Information).

### FEM

The commercial finite element package Abaqus/Explicit solver (v6.14, Dassault Systèmes) is employed to simulate the mechanical behavior of the pre‐torsion auxetic tubular structure under tension and compression. The hyperelastic Ogden third‐order model is used to simulate the 95A TPU material based on the fitting results of dumbbell‐shaped specimens (see Figure S3, Supporting Information). Due to the complexity of the pre‐torsion tubular structures, we use the tetrahedral element (C3D10M) to build the model. Each model consists of over 600,000 meshes, with a minimum mesh size of 1 mm and a maximum mesh size of 3 mm. The mesh is refined in the weak parts to ensure mesh quality.

### Nail Driving‐In Test

The nail samples are manufactured using selective laser melting technology (SLM300 printer, accuracy is 0.2 mm, Zrapid Technologies Ltd., China), and the base material is 316L stainless steel. The laser system is a fiber laser (IPG) with a wavelength of 1070 nm and a power of 200 W. In the recoating system, the layer thickness is 0.05 mm. 9 nails are prepared, with 3 of each type, to ensure the reliability of the test. The nails are driven into the wooden block by a universal testing machine at a speed of 2 mm min^−1^, which is equivalent to quasi‐static loading. Note that the cubic pine block is pre‐fabricated with round holes slightly smaller than the diameter of the nails (see Figure S9, Supporting Information), creating an interference fit.

## Conflict of Interest

The authors declare no conflict of interest.

## Supporting information



Supporting Information

Supplemental Video 1

Supplemental Video 2

Supplemental Video 3

Supplemental Video 4

## Data Availability

The data that support the findings of this study are available from the corresponding author upon reasonable request.
