# Supplementary material for: Pre‐Torsion Tubular Metamaterials: Multi‐Effect Integration for Advanced Functional Applications
Source: Adv Sci (Weinh). 2025 Aug 27;12(42):e12564. doi: 10.1002/advs.202512564 (PMC12622480; doi:10.1002/advs.202512564)
Supplement: Supplementary file 1 — Supporting Information [file ADVS-12-e12564-s002.docx]

Supporting Information

**Pre-torsion tubular metamaterials: multi-effect integration for advanced functional applications**

*Xuegang Zhang, Jianfei Yin*, Xin Ren*, Jie Wu, Yang Wang, Xingchi Teng, Wei Jiang, Dong Han, Xihai Ni, Yi Zhang, Dianlong Yu, and Jihong Wen**

X. Zhang, J. Wu, Y. Wang, J. Yin, D. Yu, J. Wen

College of Intelligence Science and Technology, National University of Defense Technology, Changsha, 410073, China

National Key Laboratory of Equipment State Sensing and Smart Support, National University of Defense Technology, Changsha 410073, China

E-mail: nmhsyjf@nudt.edu.cn; wenjihong@vip.sina.com

X. Ren, X. Teng, W. Jiang, D. Han, X. Ni, Y. Zhang

Center for Innovative Structures, Nanjing Tech University, Nanjing 211816, China

E-mail: xin.ren@njtech.edu.cn

**Supplementary Figures S1-S10**


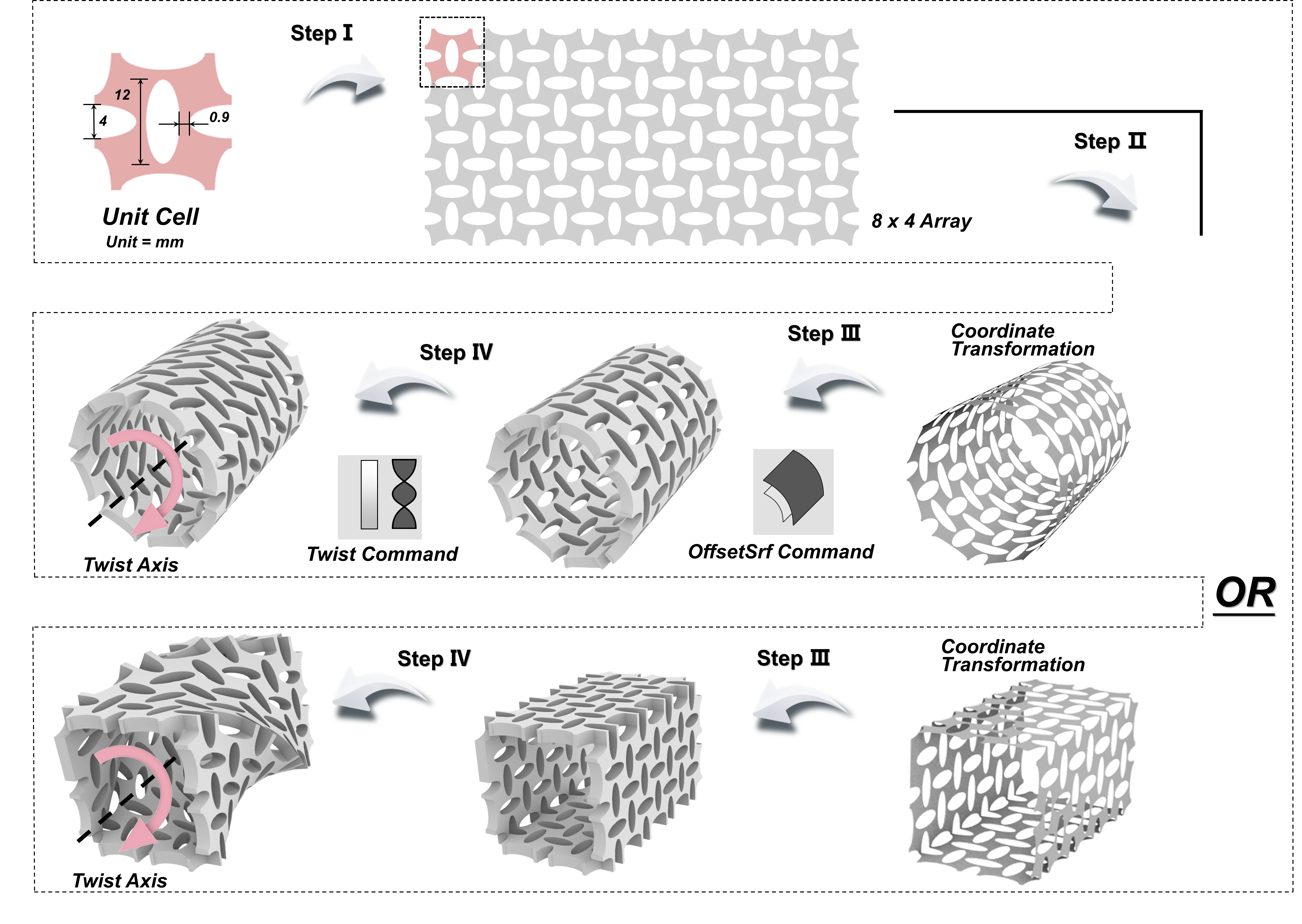


**Figure S1. Design process of pre-torsion auxetic tubular structures.** Step Ⅰ: The unit cells are constructed into a two-dimensional perforated plate by the array method (8 unit cells horizontally and 4 unit cells vertically). Step Ⅱ: The perforated plate is transformed into a tubular structure by the coordinate transformation method.^[1]^ Step Ⅲ: Use the *OffsetSrf* command in Rhinoceros 7.0 to give thickness to the tubular structure (4mm here). Step Ⅳ: Select the *Twist* command in Rhinoceros to rotate the tubular structure 90 degrees along the twist axis.


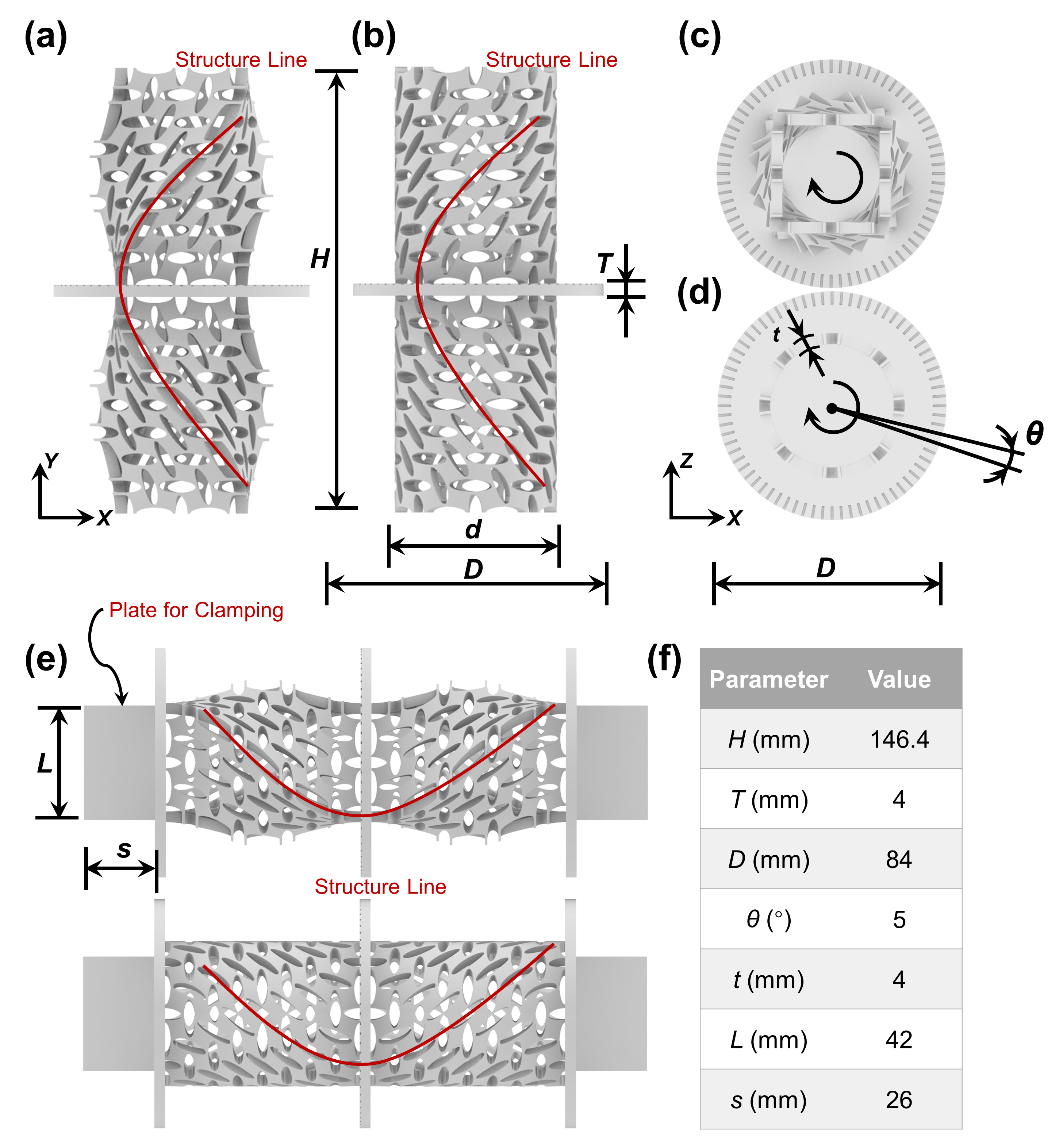


**Figure S2. Information on the geometry of the tensile and compressive members.** a,b) Dimensions of the square and circular pre-torsion auxetic tubular structures and the structure line. c,d) Top view of the square and circular pre-torsion tubular structures and the scales. e) Tensile members and structural lines. f) Detailed dimensional information.


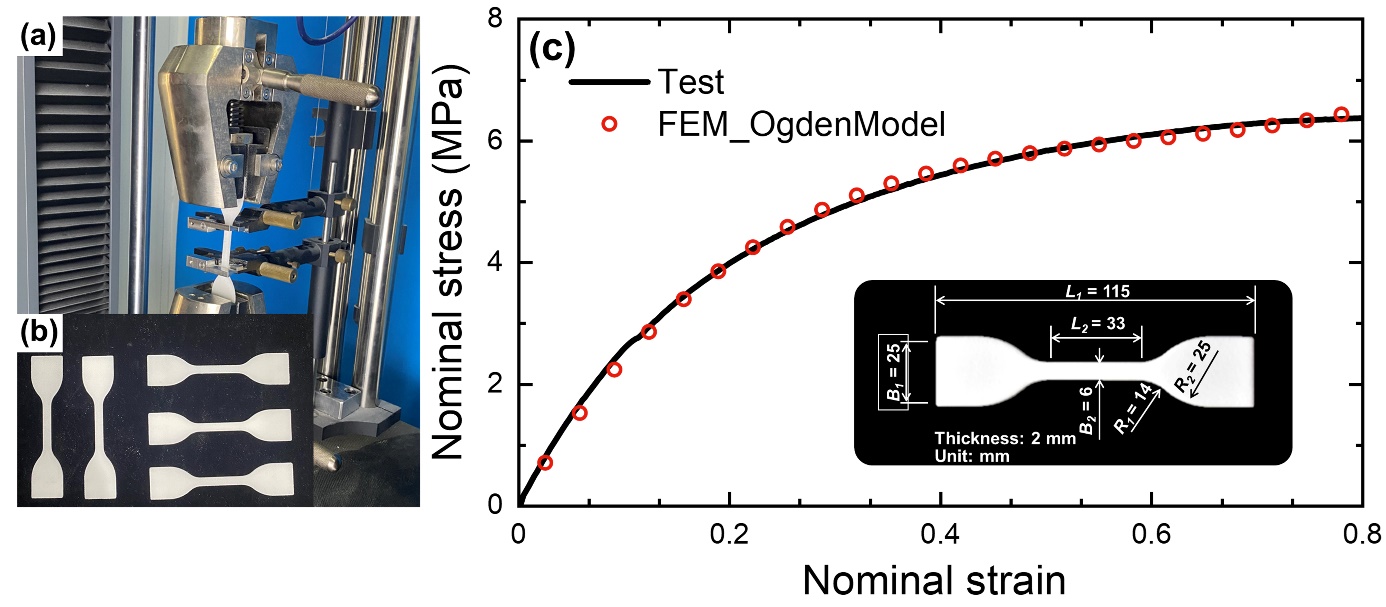


**Figure S3. Material property testing and the material model.** a) Tensile testing of TPU dumbbell-shaped specimens. b) Specimens used for testing. c) Typical nominal stress-strain curves and the fitting curve of Ogden 3rd order hyperelastic model used in the finite element analysis.^[2]^


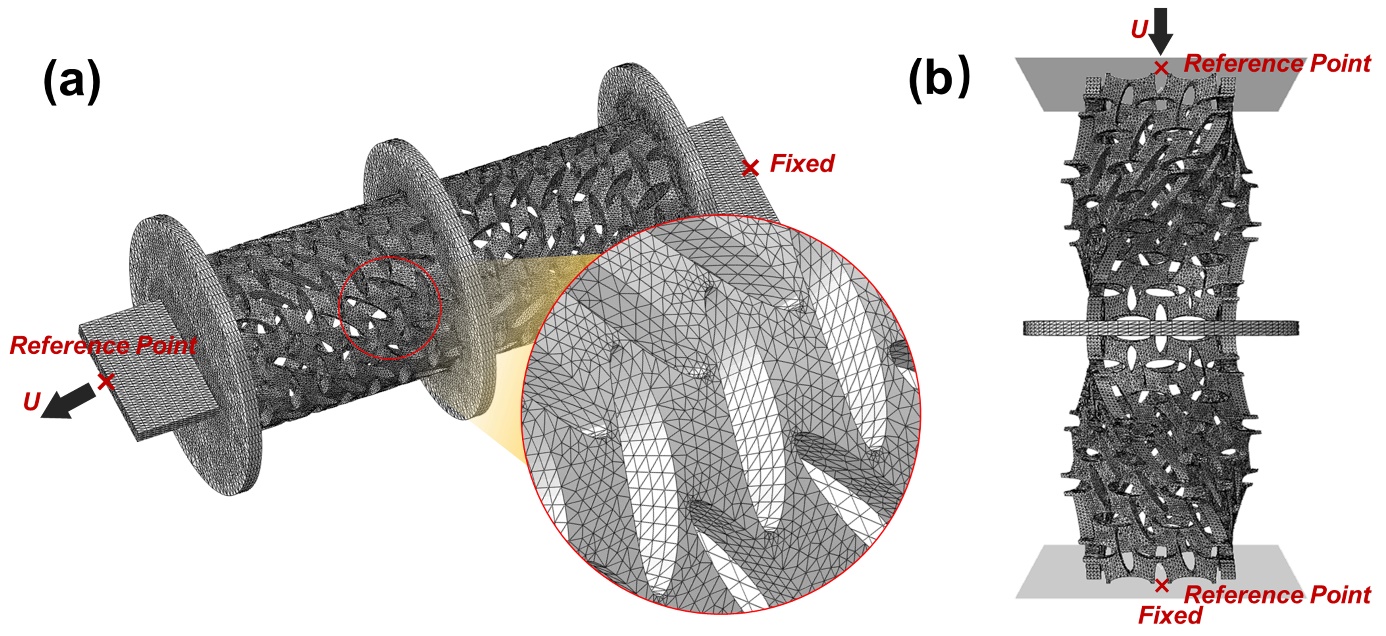


**Figure S4. Mesh and boundary condition in finite element methods.** a,b) Boundary conditions and mesh arrangement for tensile and compressive members.

In tensile members, reference points are set at both ends of the structure and coupled to the surfaces. The displacement boundary conditions are set at the reference points of one section, while the reference points at the other end are fixed. In compression members, rigid body surfaces are set at both ends of the structure to simulate the steel plate in the real case, and the boundary conditions are set on the reference points of the rigid body surfaces.


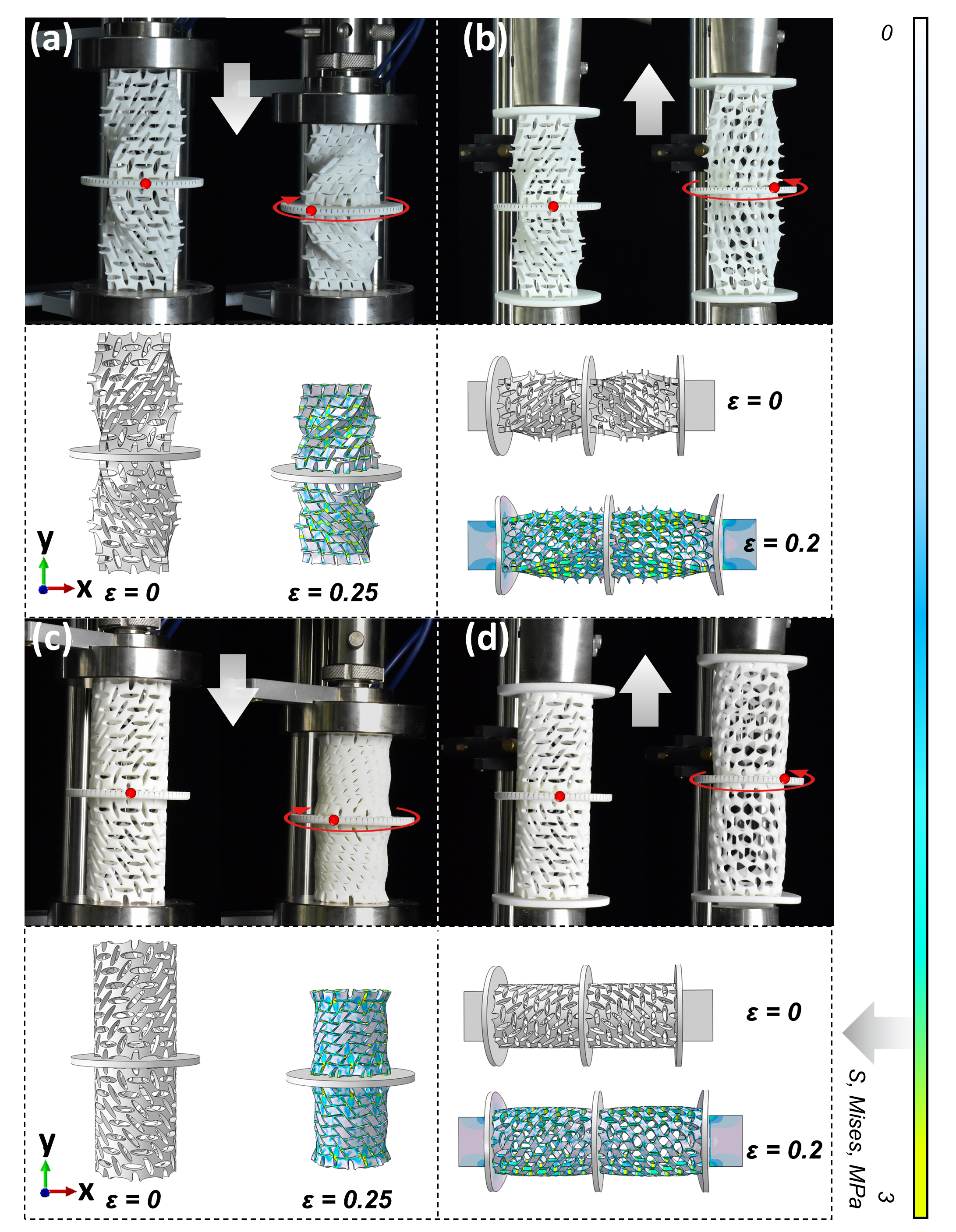


**Figure S5.** **Mechanical behavior of pre-torsion ATSs under compressive and tensile loading.** a,b) Deformation modes of SATS for 0.25 strain in compression tests and 0.2 strain in tension tests obtained from experiments and FEM. c,d) Deformation modes of CATS for 0.25 strain in compression tests and 0.2 strain in tension tests. The red mark at a fixed position of the middle disc is used to indicate the twist of the disc. The color bands in the finite element model represent the magnitude of stress.


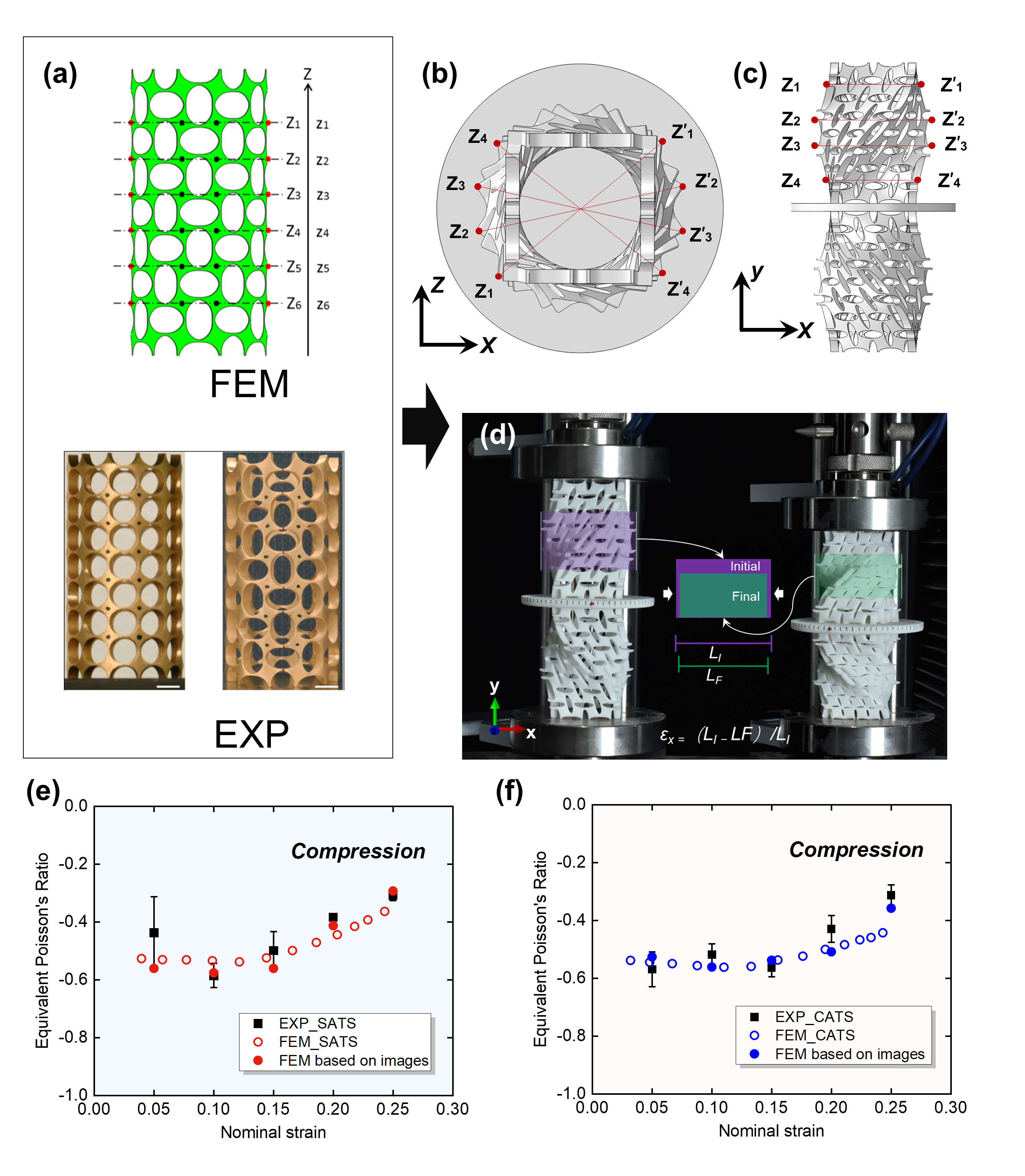


**Figure S6. Calculation of equivalent Poisson's ratio.** a) Conventional method for calculating the negative Poisson's ratio effect for tubular structures in finite element method (FEM) and experiments (EXP).^[1]^ b) Reference points for testing the equivalent negative Poisson's ratio in this paper (top view). c) Reference points for calculating the equivalent Poisson's ratio in the front view. d) An image-based computational method for determining the auxetic effect of pre-torsion tubular structures in the experimens. e) and f) The equivalent Poisson's ratio is comparatively evaluated using three distinct approaches: experimental image-based analysis, image-based finite element method (FEM), and FEM based on fixed reference points.

Due to the mirror symmetry of the structure, we choose a half-structure to calculate the equivalent negative Poisson's ratio. In this work, the tubular structure is helical, so we make some modifications to the traditional way of taking points for calculation,^[1]^ but the calculation method remains the same. The calculation method is as follows:

$\varepsilon_{i}=\frac{\sqrt{\sum_{j=x,z} \left(z_{i}^{'}j-z_{i}j\right)^{2}}}{R}-1$ (S1)

$\bar{\nu}=\sum_{i=1,2,3,4} -\frac{\varepsilon_{i}}{\varepsilon_{y}}$ (S2)

where *ε* denotes the strain between two reference points, *i* denotes the number of specific points, *j* denotes the coordinates, and *R* represents the original distance between the two reference points. *ε_y_* denotes the axial strain of the pre-torsional tube. $\bar{\nu}$ denotes the equivalent Poisson's ratio.

Note that, due to limitations in spatial measurement techniques, experimentally tracking these fixed points as in simulations is unfeasible. Therefore, we employ an alternative image-based computational method that calculates average relative displacements based on contour contraction observed in multiple image sets (**Figure S6d**). Due to boundary condition constraints on the equivalent Poisson's ratio, we select elements in the central region of the structure to calculate the transverse strain εx. Results demonstrate consistent agreement across methods for both SATS and CATS configurations (Figure S6e,f).


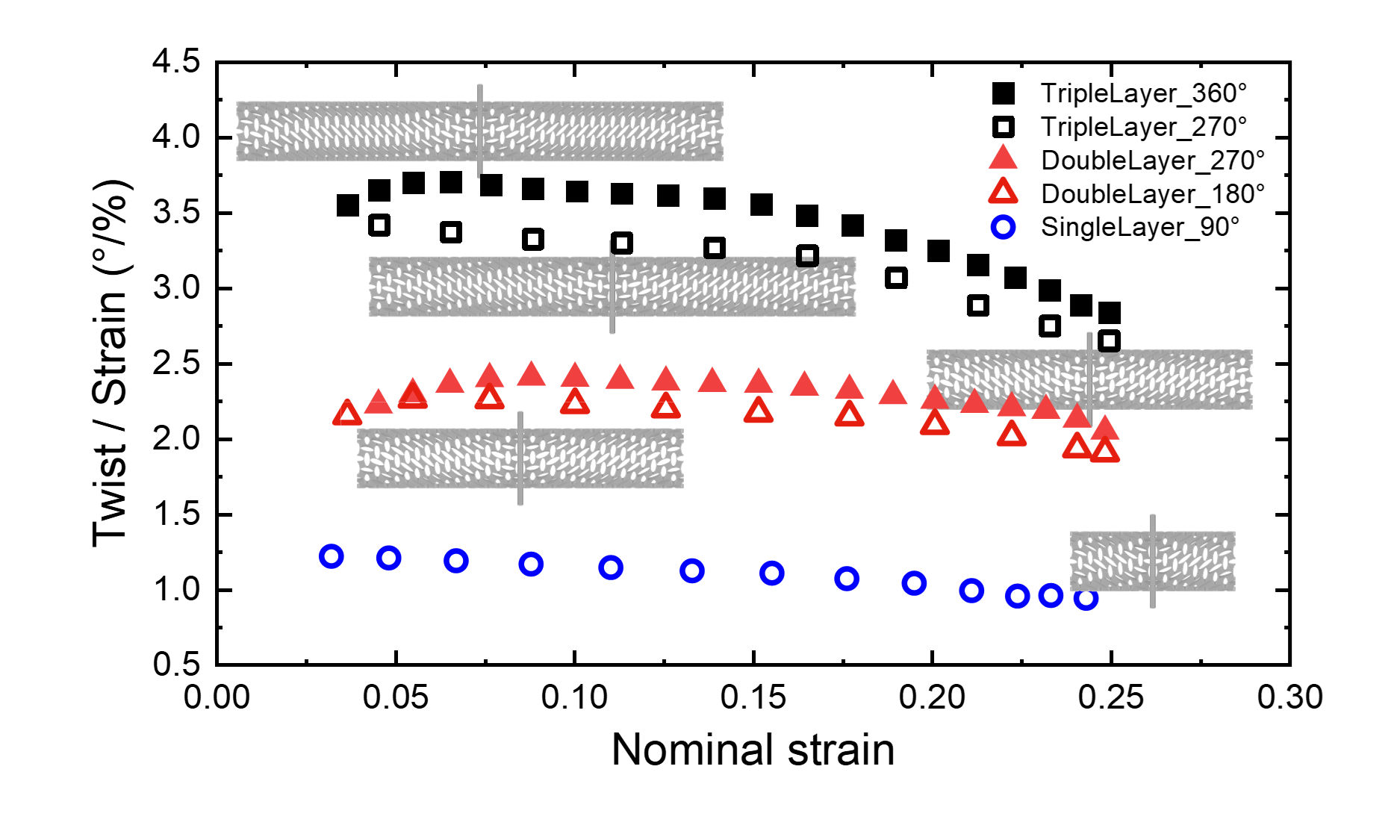


**Figure S7. Optimized design for compression-torsion effects. Pre-torsion CATSs with different structural parameters.**

The blue circle is the CATS with the pre-torsion of 90° from the manuscript. **Figure S7** shows that increasing the angle of pre-torsion enhances the compression-torsion effect, while the number of layers of the tubular structure plays a dominant role in the compression-torsion effect. When the number of layers is set to 3, the angle of compression-torsion can reach 3.7°/%. This indicates that the torsional angle can be increased significantly by increasing the number of layers of the tubular structure.


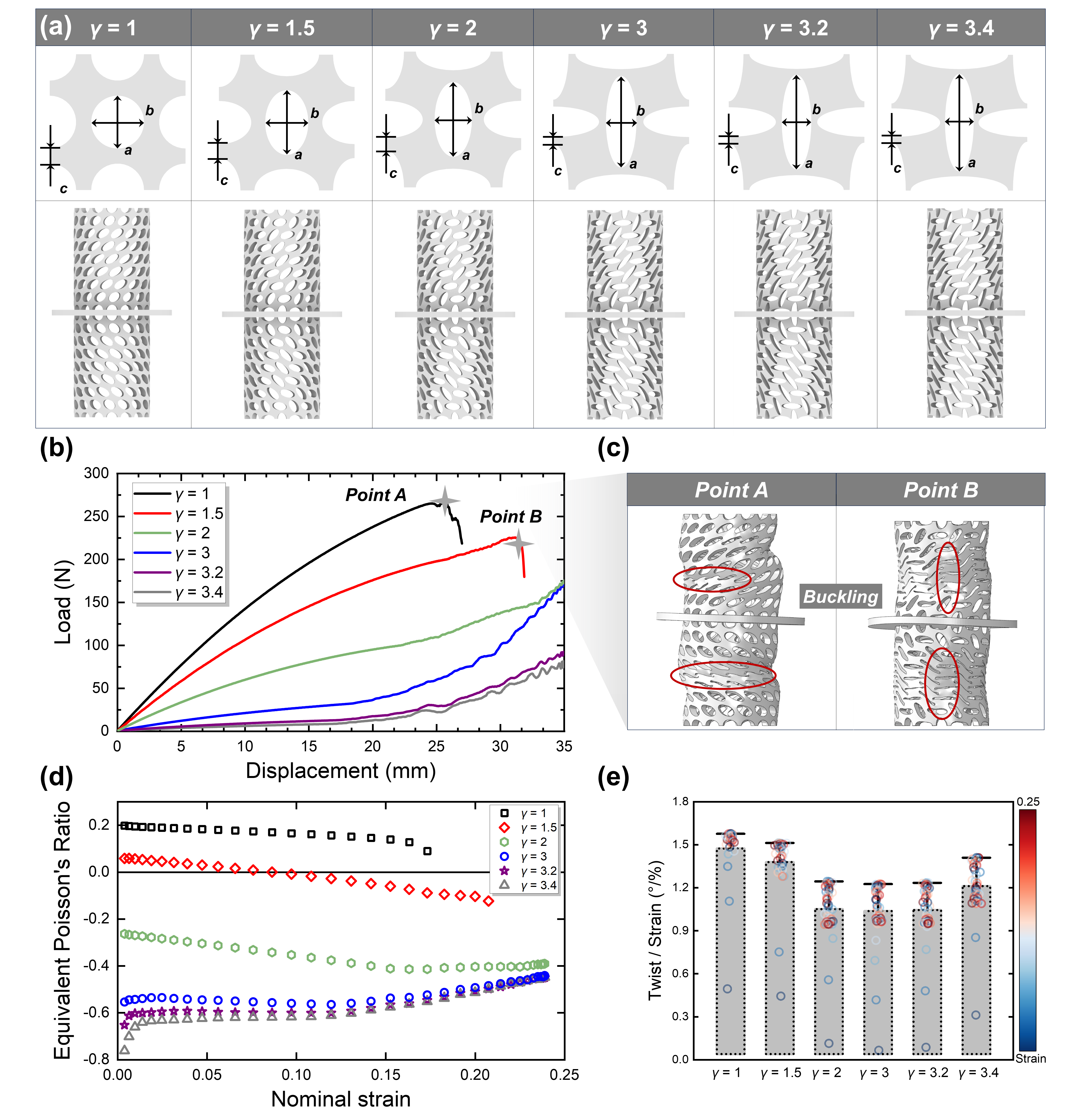


**Figure S8. Effect of the axial ratio *γ* of elliptical perforations of pre-torsion tubular structures on load-displacement curves and multi-effect integration.** a) Unit cells with different axial ratio *γ* = a/b and corresponding pre-torsion CATs. Considering the design and manufacturing boundaries, it must be ensured that c cannot be too small. Then, e is taken to be 1, 1.5, 2, 3 (in the main text), 3.2, and 3.4, respectively. b) Load-displacement curves under compression, where buckling occurs at *γ* = 1 and *γ* = 1.5. c) Deformed modes corresponding to points a and b in Fig. b. d) Variation of equivalent Poisson's ratio with nominal strain. e) Torsion angle in compression for unit cells with different axial ratios *γ*.

In **Figure S8**a, the shape of the perforation is changed by varying the ratio *γ* of the short and long axes of the ellipse. The closer the value of *γ* is to 1, the more the shape of the perforation tends to be circular. Note that we keep the areas of the unit cells the same. As *γ* closer to 1, the pre-torsion tubular structure has a higher load-bearing capacity (Figure S8b), but is more prone to buckling at the tubular wall (Figure S8c). This is because the smaller the gamma value (greater than or equal to 1), the thicker c becomes, and there is a significant increase in the load-bearing capacity of the tubular structure as the load-bearing level is mainly derived from the ligaments. However, a larger value of c leads to instability during deformation, resulting in buckling.

For the MEI, an increase in the axial ratio *γ* significantly enhances the auxetic effect, which disappears at *γ* = 1. However, the compression-torsion effect does not disappear at any value of *γ*, which suggests that our pre-torsion design paradigm can bring the stable compressive torsion effect for the structure, even if the structure is non-auxetic.


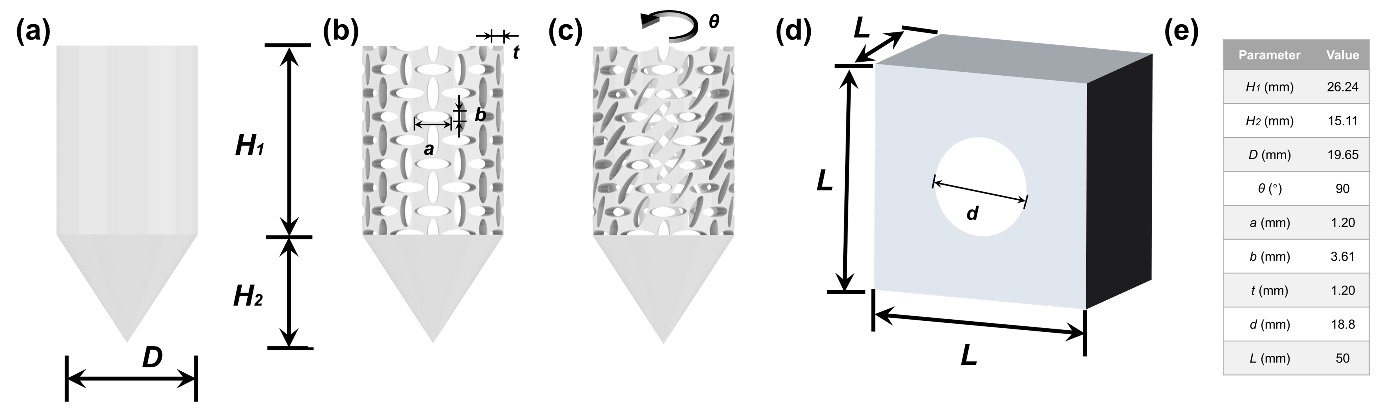


**Figure S9. Tests of nails being pushed into wooden blocks.** a) Solid nail, b) auxetic nail. (c) Pre-torsion auxetic nail. (d) Wooden block with pre-drilled holes. (e) Specific dimensional information.

**
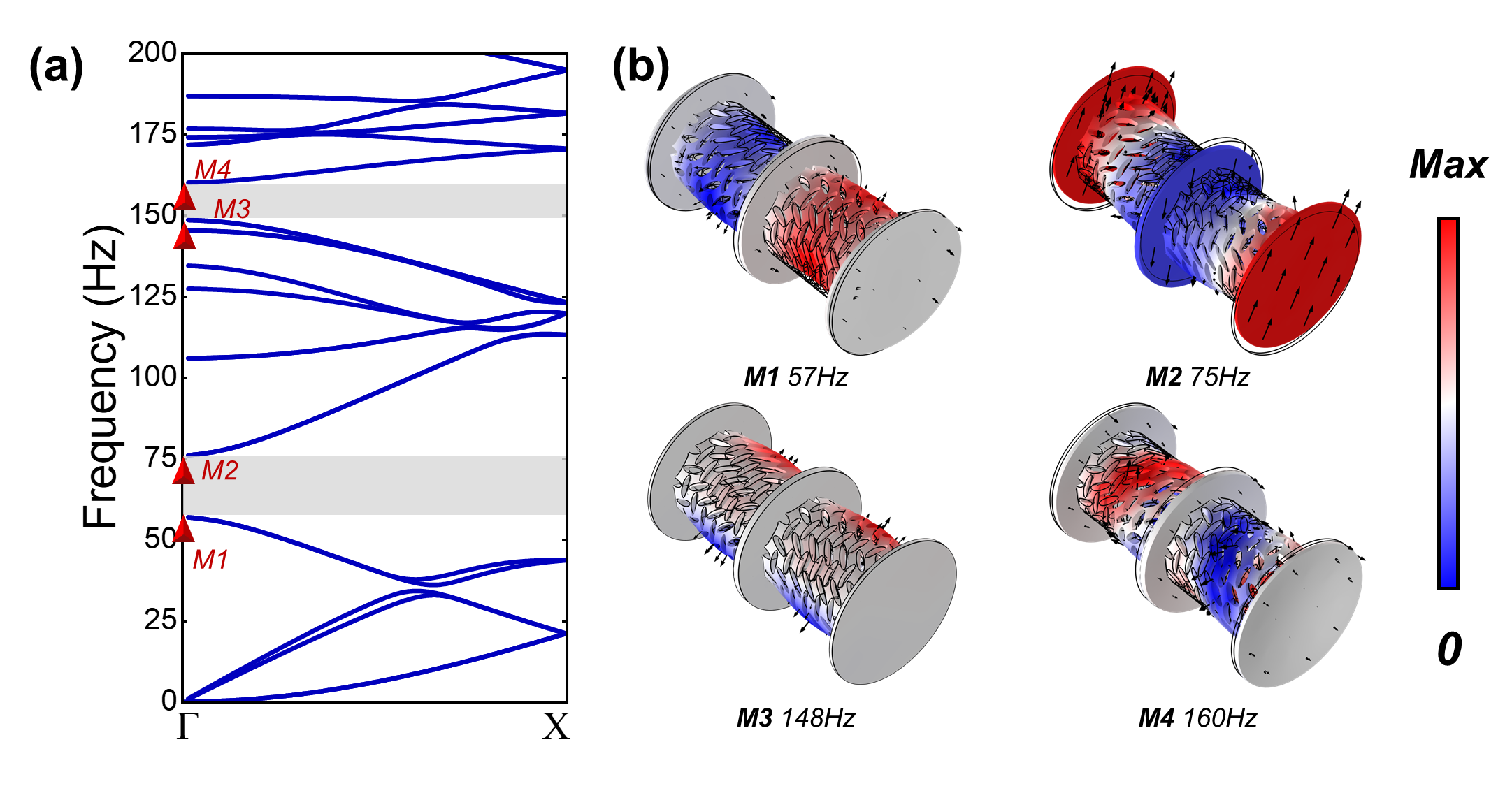
**

**Figure S10. Wave propagation characterization of pre-torsion CATS.** (a) Dispersion curve. (b) Vibration modes at the boundary of the band gap.

Three-dimensional tensor form of wave equations:

(S3)

where is Hamilton operator. The present work belongs to a one-dimensional wave problem where the wave propagates in only one direction and is assumed to propagate in the x-direction.^[3]^ In this context, **u**(**r**, *t*) becomes u(*x*, *t*). The wave equation is decoupled into 3 independent equations:

$\rho\frac{\partial^{2}u_{x}}{\partial t^{2}}=\frac{\partial}{\partial x}[(\lambda+2\mu)\frac{\partial u_{x}}{\partial x}]$ (S4)

$\rho\frac{\partial^{2}u_{y}}{\partial t^{2}}=\frac{\partial}{\partial x}(\mu\frac{\partial u_{y}}{\partial x})$ (S5)

$\rho\frac{\partial^{2}u_{z}}{\partial t^{2}}=\frac{\partial}{\partial x}(u\frac{\partial u_{z}}{\partial x})$ (S6)

where *ρ* is density, *λ* and *μ* are Lamé constants.

According to Bloch's theorem, the displacement field of a periodic structure can be expressed as:

$u(r,t)=u_{k}(r)e^{i(k\cdot r-\omega t)}$ (S7)

where **k** is the wave vector and *ω* is the angular frequency. The amplitude-modulated function$u_{k}(r)=u_{k}(r+R)$.

The dispersion curves for transverse or longitudinal waves propagating in a periodic structure can be obtained by bringing the **u** into the Equation (S4) ~ (S6).

The relationship between the wave vector *k* and the angular frequency *ω* can be obtained for periodic homogeneous materials, while it can only be solved by the finite element method for non-homogeneous complex periodic structures at present.

In **Figure S10**a, the dispersion curves of pre-torsion CATS generate two low frequency bandgaps (57-75Hz and 148-160Hz). The compression-torsion effect can be observed in the mode shapes, i.e. the coupling of longitudinal and torsional vibrations (M1 and M4 in Figure S10b). M2 is a flexural mode, while M3 is a breathing mode.

The commercial finite element software COMSOL Multiphysics 6.0 is employed to calculate the dispersion relations of the pre-torsion auxetic tubular structure. According to the Floquet-Bloch theorem, Bloch periodic boundaries are set on the upper and lower surfaces of the tubular structure. The eigenfrequencies are obtained by computing the wave vector k sweeping the simplest Brillouin zone boundary and then solving the dispersion relation of the structure. For this study, the material was set to be homogeneous, linearly elastic, and isotropic, with elastic modulus of 26 MPa, density of 1100 kg/m^3^, and Poisson's ratio of 0.495. In addition, 30 eigenfrequencies were solved with a reference value of 0 Hz.

**Supplementary Video S1 – S4**

**Video S1:** Compression and tension process of pre-torsion SATS in experimental and finite element method.

**Video S2:** Compression and tension process of pre-torsion CATS in experimental and finite element method.

**Video S3:** The process of the auxetic nail being pushed into a wood block.

**Video S4:** The process of the pre-torsion auxetic nail being pushed into a wood block.

## References

[1] X. Ren, J. Shen, A. Ghaedizadeh, H. Tian, Y. M. Xie, *Smart Mater. Struct.* **2016**, 25, 065012.

[2] R. W. Ogden, G. Saccomandi, I. Sgura, *Comput. Mech.* **2004**, 34, 484.

[3] S. Han, N. Ma, H. Zheng, Q. Han, C. Li, *COMPOS PART A-APPL S* **2024**, 185, 108298.
